# Neonatal diagnosis of isolated absence of the right pulmonary artery: a case report and review of the literature

**DOI:** 10.1186/s13052-018-0465-1

**Published:** 2018-02-20

**Authors:** Akamin Raymond, Ettore Pedretti, Giuseppina Privitera, Cristina Cicero, Giacomo Biasucci

**Affiliations:** Paediatrics & Neonatology Unit, “Guglielmo da Saliceto” City Hospital, Cantone del Cristo, 50, 29121 Piacenza, Italy

**Keywords:** Unilateral absence of pulmonary artery, Pulmonary artery agenesis, Congenital cardiovascular malformation, Transthoracic echocardiography, Thoracic MRI

## Abstract

**Background:**

Unilateral absence of the pulmonary artery (UAPA) is a rare congenital malformation often associated with other cardiac anomalies; however it may occur as an isolated lesion. Isolated absence of the right pulmonary artery is twice more frequent than that of the left pulmonary artery. Patients with isolated UAPA are usually asymptomatic at birth; thereafter they may develop a progression of symptoms such as exercise intolerance, dyspnea, chest pain, hemoptysis and recurrent pulmonary infections. As patients may remain asymptomatic or have vague symptoms, the diagnosis of isolated UAPA can be difficult to make in infancy. Indeed, most cases described in literature are adults. Due to the rarity of neonatal presentation, there is no consensus regarding the treatment of this malformation.

**Case presentation:**

Herein, the case of a two-day-old term female infant, born after uneventful pregnancy, who required a cardiological assessment for a light murmur, is reported; an echocardiogram demonstrated an isolated unilateral absence of the right pulmonary artery, confirmed by means of magnetic resonance imaging (MRI) performed 1 month after child’s birth. Besides this finding, MRI showed a slightly increased lumen and size of the main and left pulmonary arteries. The right lung was shown to be perfused by some systemic collateral arteries. In the absence of any other cardiovascular malformation, our patient did not need any treatment. As symptoms may occur later in life, a thorough clinical and cardiological follow up was immediately started. Three years later, she is still asymptomatic, showing adequate growth, without any sign of pulmonary hypertension.

**Conclusions:**

Isolated UAPA is a very rare malformation with a diverse clinical presentation. To the best of our knowledge, this is the second case of neonatal presentation of UAPA reported in literature to date. We believe that our case report supports the opinion that a prompt cardiological evaluation is needed whenever a newborn shows signs and/or symptoms of cardiorespiratory concern. Any missed neonatal diagnosis of UAPA may contribute to the later age at presentation, with resultant higher risk of morbidity and mortality and greater therapeutical difficulties.

## Background

Unilateral absence of the pulmonary artery (UAPA) is a very rare congenital cardiovascular malformation. It was first described by Frantzel in 1868 [1]. The prevalence of isolated UAPA is estimated to range from 1 in 200,000 to 1 in 300,000 young adults, but the actual prevalence of this malformation in children is still unknown. UAPA is often associated with other cardiac anomalies; however it may seldom occur as an isolated lesion, which mostly concerns the right pulmonary artery [2, 3]. Among the cardiac malformations possibly associated with UAPA, tetralogy of Fallot, atrial septal defect, coarctation of aorta, right aortic arch, truncus arteriosus and pulmonary atresia are the most frequent ones [4]. These anomalies are often associated with cyanosis, heart failure or growth retardation in the neonate and/or infant. Patients with isolated UAPA may have a different clinical presentation; usually most neonates and infants are asymptomatic, but they may develop a progression of symptoms such as exercise intolerance, dyspnea, chest pain, hemoptysis and recurrent pulmonary infections, throughout infancy and adolescence. These symptoms depend on the anatomy of the affected lung and the types of the blood vessels providing its perfusion. Due to the rarity of the disease, there is no yet general consensus regarding the treatment of UAPA. Therefore, the treatment must be tailored according to the clinical signs and symptoms, the anatomy of pulmonary artery and aortic-pulmonary collaterals, as well as the presence of associated cardiovascular anomalies and pulmonary hypertension [4, 5]. The diagnosis of UAPA soon after birth and/or in early infancy allows for early medical and/or surgical treatment, which in turn improves the effects on symptoms and outcome. Herein, the case of a neonatal diagnosis of UAPA is reported. Moreover, we provide a highlight on the data available in literature to date.

## Case presentation

**Fig. 1:**
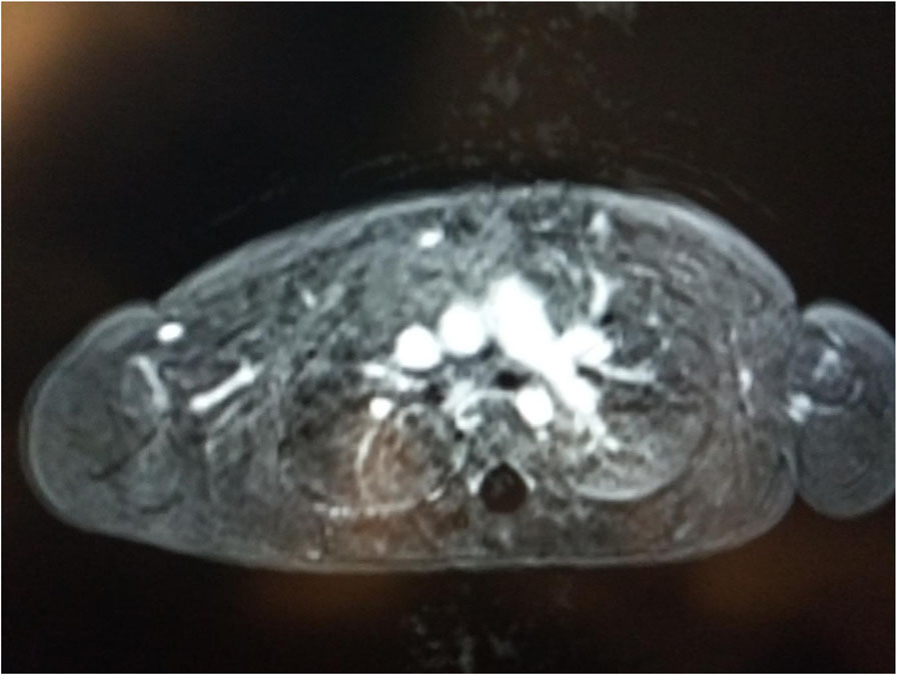
Chest MRI, at one month of age, showing isolated unilateral absence of the right pulmonary artery

A two-day-old female infant, born at term after uneventful pregnancy, was referred to our paediatric cardiology team to be evaluated for a light murmur. She was born by vaginal delivery; her post-natal adaptation was normal and auxometric parameters were adequate for her gestational age. No clinical signs of dysmorphism or cyanosis or dyspnea were evident; her peripheral oxygen saturation was 98% at room air and the heart rate was normal. On cardiac auscultation, a 1-2/VI murmur could be heard in the left upper sternal border; pulmonary auscultation as well as peripheral pulses were normal. Colour-Doppler echocardiography revealed the absence of the right pulmonary artery, whereas the main and left pulmonary arteries had normal size. The pulmonary valve was normal. There was no evidence of a patent ductus arteriosus (PDA) nor other cardiac defects except for a small patent foramen ovale. The size and function of both cardiac ventricles were normal and without ventricular hypertrophy. The size and origin of the coronary arteries, as well as the aortic arch, were normal. A physiological tricuspid insufficiency was noticed, with an estimated pulmonary artery pressure of 30 mmHg. These cardiovascular features were confirmed by echocardiographic examination, 1 week later. Suspecting an isolated unilateral absence of the right pulmonary artery (UARPA), we decided to carry out chest X-ray and MRI. The former showed normal pulmonary aeration with regular size of both lungs and a normal cardiac silhouette, the latter showed the absence of the right pulmonary artery, with a slightly increased lumen and size of the main and left pulmonary arteries (Fig. 1). Fortunately, the right lung was shown to be perfused by some systemic collateral arteries arising principally from the right subclavian artery and from the celiac trunk. MRI also excluded other heart defects and confirmed normal cardiac function.

In the light of an isolated malformation, as our child was still asymptomatic and she had regular growth rates, no medical or surgical treatment was deemed to be needed. Nevertheless, as symptoms may occur later in life, a thorough clinical and cardiological follow up was immediately started. At present, our patient is 3 years old and remains completely asymptomatic. She is a very active child and shows no sign of fatigue. She has not had any feeding difficulties and her growth has been regular. No major pulmonary infections have been reported. During her first 3 years of life, we have thoroughly monitored her pulmonary artery pressure and the right ventricular function, which have always been normal. In the light of these findings and her clinical condition, our referral paediatric heart surgeon also recommended a wait and see attitude.

## Discussion and conclusions

UAPA is a rare congenital malformation that very often occurs in association with other cardiac anomalies but in a very few cases it may present as an isolated lesion. Isolated absence of the right pulmonary artery is twice more frequent than that of the left pulmonary artery (5, 6, 7). Bockeria et al. reviewed 352 cases of UAPA reported in literature, of whom 237 (67%) were associated with other congenital heart defects [5].

The embryologic cause of UAPA is still a matter of debate. UAPA is thought to be due to a failed migration and rotation of the primitive sixth aortic arch, resulting in agenesis of the pulmonary artery. It has been observed that distal intrapulmonary branches of the affected artery usually remain intact and can be supplied by collateral vessels from bronchial, intercostal, internal mammary, sub-diaphragmatic, subclavian or even coronary arteries [6, 7].

Infants with an isolated UAPA are usually asymptomatic and may remain so until adulthood. Due to the rarity and the specific origin of this condition, the diagnosis of UAPA can be difficult at birth or during early infancy. Indeed, literature on neonatal isolated UAPA is extremely scarce. To the best of our knowledge, the case herein represents the second most precocious diagnosis of isolated UAPA reported in literature to date. The other report refers to a two-day-child presenting with episode of oxygen desaturation to 80% at 16 h of life [8]. An echocardiogram demonstrated only a single left pulmonary artery with a small ipsilateral PDA. After several hours of prostaglandin infusion and clinical stability, a second echocardiography was not able to show any discernible right pulmonary artery flow. No aortic collaterals were noted going to the right lung and no right-sided pulmonary venous flow could be seen. Right ventricular enlargement, PDA bidirectional flow, and interventricular septal flattening were all suggestive of elevated right heart pressures. Computed tomographic arteriography was suggestive of a distal right pulmonary artery (RPA) that originated from an occluded right-sided PDA. The lumen and size of the RPA could not be established as no contrast entered it. Cardiac catheterization with pulmonary venous wedge angiography confirmed the diagnosis of anomalous origin of the RPA from the innominate artery via an occluded right-sided ductus, as well as the presence of thrombus in the distal RPA. Therefore the child had to undergo surgical intervention. In the other few cases of infant diagnosis of isolated UAPA reported in literature to date, the initial sign was either cyanosis or a murmur. As the patients get older, they may develop a variety of symptoms, including pulmonary hypertension [4]. The incidence of the latter has been reported to range between 19% to 44% [9, 10]. Pulmonary hypertension is caused by an increased blood flow in the contralateral pulmonary artery, which leads to a remodelling of the arterioles, responsible for increased vascular resistance. The prognosis depends on the eventually associated cardiovascular anomalies and the degree of pulmonary hypertension [11]. Some infants presenting with heart failure have also been described [9].

In a retrospective review of 108 patients reported between 1978 and 2000, 14 patients (median age 14 yrs.; range 0.1-58 yrs) were asymptomatic. Most patients had symptoms such as frequent pulmonary infections (37%), dyspnea or limited exercise tolerance (40%). Hemoptysis occurred in about 20% of patients and high-altitude pulmonary edema was seen in about 10% of them. Pulmonary hypertension was present in 44% of the patients. Surgical procedures were performed in 17% of patients and the overall mortality rate was 7%. The aetiology of recurrent pulmonary infections is likely to be multifactorial, partly due to poor blood flow in the affected lung, ventilation/perfusion mismatch and reduced mucosal defense. Hemoptysis is these patients is caused by excessive collateral circulation [9, 10].

The diagnosis of isolated UAPA is a big challenge for neonatologists and cardiologists because of signs can be subtle and can be easily missed. There may be a systolic ejection murmur across the pulmonary outflow tract [12, 13]. The electrocardiogram is usually normal in patients with isolated UAPA. In contrast, transthoracic colour-Doppler echocardiography is a useful tool to diagnose this malformation and the eventually associated anomalies. Other investigations such as MRI or computed tomography arteriography are necessary to confirm the diagnosis and to better describe the pulmonary vasculature [12, 14].

Our patient was referred to our paediatric cardiology team because of a light murmur. In our Paediatric Unit, we perform echocardiography in all infants presenting with a murmur. At birth, other indications for ecocardiography are: arrhythmias, bradycardia, dyspnea, oxygen desaturation, family history of cardiac defects or early death, gestational diabetes, prematurity, intrauterine growth retardation, history of intrauterine fetal death, malformations and syndromic features. This gives us a higher chance to identify asymptomatic cardiac malformations as in the case herein reported. Indeed, colour-Doppler echocardiography is a relatively simple and non-invasive tool, which enables early diagnosis of many cardiac anomalies including asymptomatic malformations such as isolated UAPA. This permits early medical or surgical intervention and follow-up, thus reducing the risk of morbidity and mortality. Therefore, we are of the opinion that neonatologists should enhance their expertise in the field, becoming more familiar with the echocardiographic tecniques.

In our patient, chest MRI, performed 1 month after birth, confirmed the absence of the right pulmonary artery. Moreover, the right lung was shown to be perfused by some systemic collateral arteries arising principally from the right subclavian artery and from the celiac trunk, in the absence of any other malformation. MRI also excluded other cardiac anomalies, confirming an isolated UARPA. In some cases of UAPA chest radiography and MRI may evidence some anomalies such as ipsilateral small hemithorax, mediastinal e tracheal shift toward the affected side, ipsilateral hemi-diaphragm elevation, ipsilateral diminished pulmonary vascular markings and contralateral lung hyperinflation [14]. We recommend chest MRI to confirm the diagnosis of isolated UAPA and to identify the collateral arteries supplying the affected lung.

With regard to the adequate treatment for isolated UAPA, there is no consensus yet [15]. In case of pulmonary hypertension, treatment options include endothelin receptor antagonist (i.e. Bosentan), prostacyclin and nitric oxide. Surgical treatment may include correction of anterograde blood flow to the affected lung or lobectomy. Treatment is not required for patients without evidence of cardiopulmonary dysfunction but these patients should undergo a regular clinical follow up [2, 12, 14].

In conclusion, the diagnosis of isolated UAPA can be difficult to make in the postnatal period and/or early infancy, as it is a very rare malformation and infants may be asymptomatic. Transthoracic colour-Doppler echocardiography is a very good first line diagnostic tool, but chest MRI should be performed to establish the correct diagnosis. It also enables us to identify collateral arteries supplying the affected lung. Early diagnosis and regular follow-up are necessary in order to establish the need and timing of eventual medical therapy or surgical repair. We believe that our case report supports the opinion that a prompt cardiological evaluation is needed whenever a newborn shows signs and/or symptoms of cardiorespiratory concern. Any missed neonatal diagnosis of UAPA may contribute to the later age at presentation, with resultant higher risk of morbidity and mortality and greater therapeutical difficulties.

## Abbreviations

MRI: Magnetic resonance imaging
PDA: Patent ductus arteriosus
RPA: Right pulmonary artery
UAPA: Unilateral absence of the pulmonary artery
UARPA: Unilateral absence of the right pulmonary artery
